# Pseudoaneurysm of radial artery after heart catheterisation

**DOI:** 10.1007/s12471-021-01586-3

**Published:** 2021-06-01

**Authors:** T. H. Pinxterhuis, S. H. Hofma, C. A. da Fonseca

**Affiliations:** 1 Department of Cardiology, Medical Spectrum Twente, Enschede, The Netherlands; 2 Department of Cardiology, Medical Centre Leeuwarden, Leeuwarden, The Netherlands

**Fig. 1:**
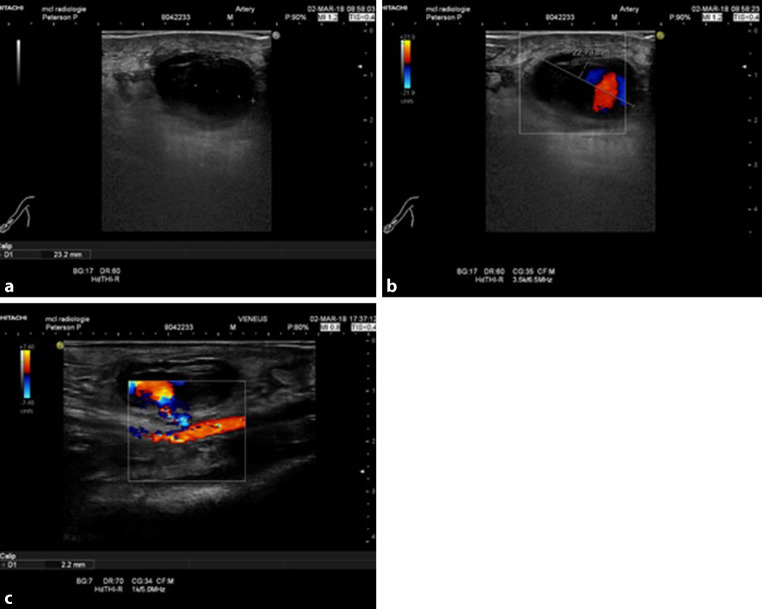
**a** Doppler ultrasonography showing pseudoaneurysm of right radial artery. **b** Echography with colour. **c** Flow from radial artery into pseudoaneurysm

A 71-year-old male underwent an elective percutaneous coronary intervention with a 5-French radial artery sheath. Hours after the procedure, he developed a painful swelling at the insertion point of the sheath. Echography showed a pseudoaneurysm of the right radial artery (Fig. 1). Thrombin injection was deemed impossible because of the short neck of the aneurysm and therefore surgical excision was performed.

Pseudoaneurysm is a known complication of arterial catheterisation. The incidence of pseudoaneurysm after femoral artery catheterisation is 0.2%–3% [1]. Risk factors are obesity, multiple vessel punctures, local vessel sclerosis, hypertension and diabetes mellitus [2]. Therapeutic options include conservative treatment, thrombin injection and surgery. Surgical intervention depends on the presence of pain, size, growth, limb ischaemia, infection and nerve compression. Pseudoaneurysm of the radial artery is extremely rare (incidence 0.009%) [3]. More than 50% of patients with a pseudoaneurysm undergo a surgical intervention [3].
